# The New York Physician’s Mutual Aid Association

**Published:** 1892-04

**Authors:** 


### THE NEW YORK PHYSICIANS' MUTUAL AID ASSOCIATION.

We have heretofore directed the attention of our readers to the beneficent work of this medical association, which admits to its membership any regular physician who is a resident of New York State, under fifty years of age, and in good health. The annual report for 1891 is before us. The most striking feature therein contained is the fact that over $83,000 have been paid to the families of deceased members since its organization, twenty-four years ago.

The steady growth of the Benevolent (permanent) Fund, the interest of which is available for aiding poor and distressed members, by loans or donations, is another very gratifying evidence of the wise administration of the Board of Trustees, which, it should be stated, has always served the society without any compensation, except the satisfaction of aiding the unfortunate in our ranks.

The growth of the membership has been rapid of late years, and there are now over one thousand names upon the roll, hence the Trustees will very soon be able to pay $1,000 upon the death of a member. We believe it is a duty we owe each other as members of the profession to unite with the Association,—those who are prosperous, for the privilege of assisting the less favored, and those who may be less fortunate, to provide for those who may be dependent upon them for support.

The expense of membership is small, not over $15 annually.

The number of members in Buffalo alone should be at once increased to at least 100. Application may be made through Dr. W. G. Gregory, No. 530 Main street, Dr. S. A. Dunham, No. 72 West Chippewa street, or to the Secretary of the Association, Dr. J. E. Nichols, 456 Lexington avenue, New York City.

